# A Qualitative Evaluation of the Pain Management VA-ECHO Program Using the RE-AIM Framework: The Participant's Perspective

**DOI:** 10.3389/fpubh.2020.00169

**Published:** 2020-05-15

**Authors:** Sherry Ball, Krysttel Stryczek, Lauren Stevenson, Rene Hearns, David H. Au, P. Michael Ho, David C. Aron

**Affiliations:** ^1^Louis Stokes Cleveland VA Medical Center (LSCVAMC), Cleveland, OH, United States; ^2^VA Puget Sound Health Care System, Seattle, WA, United States; ^3^Rocky Mountain Regional VA Medical Center (RMRVAMC), Aurora, CO, United States

**Keywords:** pain management, continuing education, tele-mentoring, primary care, specialty care

## Abstract

**Introduction:** Veterans frequently seek chronic pain care from their primary care providers (PCPs) who may not be adequately trained to provide pain management. To address this issue the Veterans Health Administration (VHA) Office of Specialty Care adopted the Specialty Care Access Network Extension for Community Healthcare Outcomes (VA-ECHO née SCAN-ECHO). The VA-ECHO program offered training and mentoring by specialists to PCPs and their staff. VA-ECHO included virtual sessions where expertise was shared in two formats: (1) didactics on common pain conditions, relevant psychological disorders, and treatment options and (2) real-time consultation on patient cases.

**Materials and methods:** VA-ECHO participants' perspectives were obtained using a semi-structured interview guide designed to elicit responses based on the RE-AIM (reach, effectiveness, adoption, implementation, and maintenance) framework. A convenience sampling was used to recruit PCPs and non-physician support staff participants. Non-physicians from rural VHA sites were purposively sampled to gain diverse perspectives.

**Findings:** This qualitative study yielded data on each RE-AIM domain except reach. Program reach was not measured as it is outside the scope of this study. Respondents reported program effectiveness as gains in knowledge and skills to improve pain care delivery. Effective incorporation of learning into practice was reflected in respondents' perceptions of improvements in: patient engagement, evidenced-based approaches, appropriate referrals, and opioid use. Program adoption included how participating health care systems selected trainees from a range of sites and roles to achieve a wide reach of pain expertise. Participation was limited by time to attend and facilitated by institutional support. Differences and similarities were noted in implementation between hub sites. Maintenance was revealed when respondents noted the importance of the lasting relationships formed between fellow participants.

**Discussion:** This study highlights VA-ECHO program attributes and unintended consequences. These findings are expected to inform future use of VA-ECHO as a means to establish a supportive consultation network between primary and specialty care providers to promote the delivery evidence-based pain management practices.

## Introduction

Most Veterans receive primary care relatively close to home at Veterans Healthcare Administration (VHA) community-based outpatient clinics (CBOCs) where access to specialty care, such as for chronic pain, is greatly needed but limited. Although pain is documented in approximately 50 percent of primary care visits at VHA facilities (1), primary care providers (PCPs) are not adequately trained to provide pain care (2). In response, the VHA has promoted initiatives designed to train and support PCPs working in CBOCs to provide chronic pain care.

The specialty care access network extended community healthcare outcomes (VA-ECHO; née SCAN-ECHO) program is an initiative implemented in 2010 to improve specialty care access, This initiative focuses on VA sites with limited specialty care resources. The program, adapted from Project ECHO® (3), offers PCPs training on the delivery of routine specialty care at their respective CBOCs. Training sessions are led virtually by a multidisciplinary specialty care team typically located at an urban VHA medical center (hub site) and simultaneously attended via teleconferencing by 10 or more PCPs from multiple VHA spoke sites, either CBOCs or rural VHA medical centers. Sessions include a didactic presentation on a series of specialty-related topics, live consultation with a specialist team, and the opportunity to learn from fellow PCP participants' consultations. At each session a PCP typically presents a case and obtains feedback from the specialty team. Participants have the opportunity to ask follow-up questions in real time and to discuss treatment options and challenges with other participants. When needed, the PCP can obtain follow-up consultation(s) at a future session(s). Sessions are recurring with different cases and topics covered at each session. Hub sites were able to design their own program, curriculum, and participation expectations. Presentation topics fit into the following categories: pain etiology, comorbidities, psychology (Post Traumatic Stress Disorder, anxiety, etc.), medications etc., as well as, guidelines on treatments, evaluation, and management. Over time relationships between specialty and primary care providers can be fostered through regularly scheduled teleconferencing sessions. Our objective was to evaluate this program to inform the expansion and implementation of pain management.

## Materials and Methods

This evaluation used qualitative methods to explore program participants' experiences with providing pain care. This work was approved, supported, and funded by the VHA Office of Specialty Care and the Office of Rural Health as an evaluation study. Our goals included understanding and improving implementation of VA-ECHO. This work was deemed quality improvement and exempt from Institutional Review Board oversight. The Louis Stokes Cleveland Department of Veterans Affairs Research and Development Committee waived the requirement for the ethical approval for this study because the project was designed and implemented for the purposes of improving internal VA processes in support of the VA mission in accordance with the national legislation and the institutional requirements.

Employee unions reviewed and approved the interview guide before initiating participant recruitment. Participation was voluntary and responses are kept anonymous.

### Participants and Setting

We obtained attendance data from four active VA-ECHO Pain Management programs (Hub sites). Two of the four hub sites were excluded due to incomplete participation data, such as missing participant contact information. The remaining two hub sites (Hub A and B) were selected for participant sampling based on confirmation of attendee's name, VHA email, spoke site location in VHA databases. Programs were designed to include a case presentation at each session. One site used a video-teleconferencing format and the other an audio-teleconferencing system with a chat box for entering written comments or questions. Both held regular weekly sessions (with some breaks, i.e., for holidays) spanning over at least 1 year. We sampled past and current VHA PCPs, ancillary providers, and clinical support staff who attended programs offered by the selected Hub sites. Participants' spoke sites were identified, including urban and rural VHA Medical Centers and CBOCs, and participants from rural facilities (serving a patient population composed of 50 percent or greater rural Veteran population according to VHA administrative data) and non-physicians were purposively sampled to obtain diverse perspectives. Participants with non-VHA email addresses (i.e., university affiliates) and those who were no longer employed with the VHA were excluded.

### Data Collection

We contacted 537 program participants by VHA email to participate in a telephone interview. Twenty-three contacts responded to decline the interview invitation, citing lack of interest in participating in the interview, not having time to participate, or incorrect information about their program participation. Six contacts accepted the invitation but did not complete scheduling. All other invitations were assumed to be declined if contacts were non-responsive. From May 2018 through September 2018, we completed 26 interviews with program participants (Table 1), including 20 females and six males. Fifteen respondents practiced at a rural facility. Three respondents had experience with both Hub programs. Six interviews were completed with respondents reporting limited experience with the program, including those who discontinued attendance after a few sessions or reported no attendance. The qualitative team ensured reflexivity by acknowledging and identifying their assumptions and existing knowledge about VA-ECHO pain management program during regular team meetings (4).

**Table 1:** Program participant interviews by provider type.

**Provider type**	**Program participants**	**Limited/No program participation**
Physicians	7	3
Physician assistants	2	0
Nurse practitioners	3	0
Registered dieticians	2	0
Pharmacists/Clinical pharmacists	4	1
Nurses	0	2
Other non-physicians	2	0

A semi-structured interview guide (see Supplementary Appendix) informed by RE-AIM (5) and past evaluation work (6, 7) was used to gather detailed descriptions and examples of respondents' experiences with the VA-ECHO pain management program. The interview guide included open-ended questions and semi-structured probes for uniform data collection of key topics and allowed exploration of unanticipated themes generated by respondents. Probes using respondents' verbatim words or phrases were used to elicit additional details and to ensure understanding of the respondent perspective rather than relying on the team's assumed knowledge. The interview guide was adapted according to the respondent's role and experience with the program (8, 9). For respondents with limited or no experience with the program, interviews focused on barriers and facilitators to program attendance, and access to pain management consultation and resources. Interviews lasted approximately 30 min.

Interviews were conducted by trained and experienced qualitative interviewers (SB, LS, and KS). All three female interviewers are research scientists who collectively have over 20 years of VHA health services and quality improvement experience and have been working together on the evaluation of specialty care initiatives for over 4 years. Interviews were audio-recorded and transcribed verbatim by Transcription Outsourcing, LLC. ATLAS.ti® software was used for data management, coding and analysis. We collected and analyzed data concurrently. Data were analyzed using iterative deductive content analysis applying *a priori* codes (Table 2) using RE-AIM (5) and inductive content analysis using open and unstructured coding to capture data that did not fit into *a priori* categories. The qualitative team met weekly to review data and reach consensus on interpretation of themes and findings. Findings were aggregated across sites and respondent types for each RE-AIM domain.

**Table 2:** RE-AIM a priori constructs.

**Domain**	**Operational definition**	**Findings**
Reach	The number of patients expected or shown to reap the main benefit from the program	Beyond the scope of this paper
Effectiveness^1^	How participation in the program improved the attendees' ability to provide quality patient care	Attendees reported gaining multidisciplinary knowledge and increased job satisfaction, improved self-efficacy in communicating with, providing care for, offering resources to patients, reduced opioid prescribing, and referring patients with chronic pain
Adoption	A description of the program attendees, their roles, and how they learned about the SCAN-ECHO program	Attendees were PCPs and support staff in primary care; informed about program from local leaders or from professional conferences
Implementation	How *spokes* sites implemented SCAN-ECHO at their sites, including barriers and facilitators to participation How *hub* sites organized the content and format of the SCAN-ECHO program and sessions	Participation in the program was possible when local leadership supported session attendance by allowing schedules to be blocked and offering continued education credit Hub sites implemented programs using different delivery platforms (video and/or audio only) and with different attendance expectations (regular attendance expected or no expectations). Didactic sessions covered a similar range of topics
Maintenance	How participants continue to use knowledge and skills obtained from the SCAN-ECHO program	Attendees continued to learn from program by continued attendance or continued interaction with fellow attendees or program leaders

1 *Effectiveness measured at PCP level, not patient outcomes*.

### Findings

#### Reach

The goal of the VA ECHO pain management program was to improve pain care for patients. Our work focused on how program participants and how they gained knowledge to improve patient care through learning and mentorship. Thus, Reach, defined as the number of patients expected or shown to reap the main benefit from the program, was beyond the scope of our work.

#### Effectiveness

Consistent with a focus on the provider level and not patient outcomes, effectiveness is reported as the extent to which providers gained skills and knowledge in pain management. Most respondents reported that the program provided a level of multidisciplinary knowledge that was not included in prior medical education and training. This training improved their knowledge of treatment options and their ability to communicate with specialists and to make better and more timely referrals. Many respondents reported having few local pain resources (i.e., pain specialist or alternative medicine providers) to address their questions before participating in the program. Respondents noted a reduction in opioid prescribing with VA-ECHO participation.

*It was kind of changing the conversation and changing the approach, how we approach, you know, somebody with comorbid psychological conditions that are overlapping, … it was just really a plethora of information being provided that I can really apply in dealing with my patients that have pain*.

Nurse Practitioner, Rural Medical Center

*I think patients are getting referred more quickly to the specialist that's going to give them the most benefit for what they have wrong with them. I think the physicians that have been through it make decisions faster about these things. I think also, it stops so many inappropriate consults that other physicians have to deal with, whether they wind up seeing the patient or not, they still have to review the consult and make some determination. And that takes up people's time needlessly. So, I think it's helped on many levels*.

Physician, Urban Medical Center

…*and I found it to be very helpful facility wide. I noticed just changes in prescribing patterns in the physicians that participated, I noticed the referrals to pain management were more appropriate, some patients had more of a workup, the providers were, actually, trying to figure out the etiology of their pain before sending them somewhere. They wound up going to the more appropriate specialist*.

Physician, Leadership, Urban Medical Center

Many providers reported increased empowerment and confidence in their ability to provide pain care. The connection of providers through VA-ECHO made them feel they are not facing the challenges of providing pain care alone. Some respondents commented that participation improved their job satisfaction.

*I really feel like I've operated independently on this island with no assistance for a while and very uncomfortable and frustrated and so forth with no direction. Having now gotten to participate in SCAN-ECHO, …I'm starting to see the benefits of it, just being able to talk about it week after week after week. You develop so much more of a comfort in here that other people are facing the absolute same things you face in your clinics every day. And, you are able to gain insight from other providers on how they do things, and why they do that. It's just started to allow me to focus on pain as something other than a pain to myself as a provider*.

Physician, Urban CBOC

*It has helped me dramatically deal with the tremendous sea change in practice and practice expectation and what is good practice. And it helped me to deal with resistance in patients and to deal with resistance in VA providers, and VA administrators saying “that's not the way we do it, that‘s not gonna' work”, sort of the consistent messaging became my voice. It was so important for me to learn. I am really grateful [*for this opportunity*] for letting me learn and letting me fail a little*.

Clinical Pharmacist, Rural Medical Center

Learning how to talk to patients specifically about use of opioids was emphasized as a skill that was valued and well-suited for this platform. However, another provider mentioned that when they stop opioids, or refuse to provide opioids for pain management, patients sometimes seek care elsewhere and don't return to that VHA provider.

…* I don't dread my pain patients if they come in. You run the list in the morning and there's a sense of dread to some degree on some folks. So, I feel like that's less, I don't feel that way as much, there's still a few, but I feel like I have more control, or more of an idea of how to, how to even talk to them and address their pain treatment*.

Physician, Urban CBOC

*There's been some patients who basically some stop coming once you stop their narcotics. Now, that's not surprising; that's happened to all of us. I don't know if that's unintended. I think a small portion of patients, that will be the case, they will go somewhere else*.

Physician, Urban CBOC

#### Adoption

Respondents at spoke sites included a variety of roles and clinical backgrounds, including physician and non-physician PCPs (i.e., physician assistants, nurse practitioners) and direct care support staff. Who participated depended upon the current needs of primary care at spoke sites. Spoke site leadership directed and encouraged staff to attend VA-ECHO sessions. Respondents reported they were expected to bring information and skills back to their site to provide new knowledge or better support for existing services.

*The plan was to have one provider from each of the CBOCs. We especially wanted the more rural CBOCs* [that] *we felt were the most important, because we had a lot more issues with opioid prescribing in those areas and not much support. The patients would have to come all the way to the main facility to see a pain management specialist. So, we made sure that the furthest away CBOCs each had a physician that attended, that was approved to attend it. Chief just blocked out that time in their schedule for the entire year and everyone knew; it's happening, and everything was fine*.

Physician, Urban Medical Center

*Most of the primary care don't participate unless their staffing a case, but because that [*presenting a case*] was made part of my job per say. It just is*.

Non-physician, Urban Medical Center

#### Implementation

Limited time was reported as a barrier to attendance. However, supervisory and institutional support were identified to mitigate this barrier. Support was leveraged from Chiefs of Staff and other supervisors to encourage providers at spoke sites to attend. Many respondents reported that having their schedules blocked during VA-ECHO sessions was the key for consistent attendance. Respondents reported they were more likely to attend when continuing education credit was offered or when VA-ECHO sessions were scheduled at times when providers were more likely to be available such as during lunch time in their respective time zone.

*We have a couple, one or two, primary care providers that participate regularly and come down regularly because we can get CEUs from them as well*.

Non-physician, Urban Medical Center

One spoke site discontinued participating in the program after realizing the program did not fit their needs. Some non-prescribers felt the program was less suited to them in that a large portion of the program content was related to opioid prescribing practices.

*It [*attending VA-ECHO*] didn't work out for us and I wish there was a little bit less focus on opiates, you know, not using opiates, but the format I think of the SCAN remote videoing in and it's a good, I like it. I think it could be really great if rolled out well, led by somebody with kind of a broader view of the landscape*.

Physician, Rural Medical Center

*If they [*VA-ECHO sessions*] are in those block times, I can participate and make efforts to meet with some of my peers, but usually our pain scope is very limited. For example, we are limited to offer opioids. Opioids are restricted for my scope, but we can refer on pain clinic*.

Nurse Practitioner, Urban Medical Center

One Hub site used video teleconferencing with no more than 15 attendees per session and the other Hub site used an audio only format allowing 100 or more to participate in a session. For some, utilization of a video format allowing attendees to see each other during each session was an initial deterrent, but after attending a few sessions participants began enjoying the video component and saw the advantage of being able to see fellow participants. Discussion was a key component of both formats.

*It was really it was nice to see the team, the pain management providers and get to know their faces and be able to ask them questions directly. Especially as a new provider, totally new to the VA it allowed me to be a little bit more comfortable with that particular specialty*.

Physician Assistant, Urban CBOC

*I think that there's less of a group discussion on the Adobe platform [audio only] in some respects. There's a larger participation because it's not a direct video conversation. A lot of people can certainly put in their two cents on the chat*.

Non-physician, Rural Medical Center

In addition to the didactic component of the VA-ECHO sessions, case presentations were also integral. Although only a few of our respondents had presented a case at VA-ECHO, respondents felt that understanding and observing how others treated chronic pain in the VHA helped them learn how to incorporate chronic pain treatment into their practice. Respondents with experience practicing outside the VHA mentioned differences in treatment practices present between the private sector and VHA, adding that VA-ECHO helped them understand VHA practices and available resources.

*I was able to learn how the pain management providers thought through chronic pain at the VA, just because it's very different here at the VA than outside the VA. And so, it was good to see the cases that presented were consistent with your treatment plan. It was good to see the consistency of care and how I guess intentional they were with each of their patients. It was also helpful to see what resources the VA had to treat pain, more than just medication like the chiropractic care and the alternative modalities and things like that*.

Physician Assistant, Urban CBOC

#### Maintenance

Many respondents continued to consult with fellow participants and specialists regarding pain care after completing the 1 year program. Some respondents continued regular or intermittent participation beyond a year in one or more Pain VA-ECHO programs depending on providers' interest, perception of learning potential, and schedule availability. One respondent reported their participation ended despite their interest to continue because of perceived competing priorities for the use of clinical time resulted in the loss of institutional support for program attendance.

Respondents also expressed improved job satisfaction. A desire to have and be a part of a support system where participants could contribute to a greater effort to improve pain management on a national level project was cited as a motivating force to attend the program.

*I think that you know we really felt the program to be enormously helpful here. All the providers that went through it enjoyed it and learned things and looked forward to it every week. It was good. It built comradery among people that otherwise wouldn't interact, other than “why did they send me that consult? That's not appropriate.” So, it's really good. There was no level that it wasn't a positive thing. It was just positive across the board. It was great. Everyone should do it*.

Physician, Urban Medical Center

…*sometimes in rural clinics or even in urban clinics where people are so busy they hardly ever leave their offices, it can be great to work together on a case or think through a case in a safe space; you're not with someone who is your boss or someone who is your superior*.

Clinical Pharmacist, Rural Medical Center

Continued contact with other participants and specialists to ask questions rather than sending numerous consults is another way some individuals hoped to continue to use what they learned in the program. Relationships between fellow participants and with specialists developed during participation, facilitated providers utilizing their connection to other provider participants for advice on difficult cases, or reaching out to specialists outside of the VA-ECHO sessions for advice.

*I can call* [specialist from program A] *or* [B] *or whoever you know if I've got a patient issue, because I don't have a provider here right now, so if I've got an issue that I really need* [to be*] addressed, and I can't find somebody around to do* [it]*, then I can call* [program A] *and say ‘Hey* [specialist X]*, can you give me input on this? Do I need to send this emergently somewhere or do I need to do whatever?' They're not just available during the SCAN, the video SCAN time. It's a long-term relationship*.

Non-physician, Rural Medical Center

## Discussion

Utilization of the RE-AIM framework with qualitative inquiry (5) highlighted aspects of the VHA pain management VA-ECHO program consistent with other published evaluations. These findings support other studies in which respondents reported program *effectiveness* as improved referrals to specialists, skills, and knowledge of available resources and treatment options for Veterans with chronic pain (10–13). Many respondents reported that improved confidence enhanced their ability to talk to patients about their pain care. Respondents who were prescribers described increased comfort in reducing the use of opioids similar to other studies and as shown quantitatively (14–17). Some reported improved job satisfaction. This and former examinations of *adoption* identified program participants as primary care team members from a range of health care provider positions who critically needed and wanted this training (1–11, 14). The primary barrier to program *implementation* was time constraints, as has been noted in other studies (18). Aspects of program *implementation* were consistent with other programs including weekly didactics. Case discussions on highly relevant topics with the multidisciplinary team, and fellow PCPs facilitated learning and provided support to clinicians who otherwise had few interactions with other VHA PCPs or pain specialists (14, 19). Respondents noted that *maintenance* of knowledge and skills occurred through continued relationships and contact with the multidisciplinary specialty care team members and fellow clinicians. Continued widespread implementation of this program is likely to continue only when the benefits of participation to patient care are balanced with the pressure to see patients and do not conflict with patient care duties.

While efforts are underway to ensure fidelity to the original Project ECHO® model and to provide guidance for consistent replication (20), implementation of the VA-ECHO program and other ECHO-like models (21) is not standardized. Allowing individual VHA hub sites to design their own programs may produce a stronger program with more local buy-in but can make comparisons of programs challenging. Due to the limited availability and time of PCPs, future studies need to address necessary and sufficient components of the VA-ECHO training model. Efforts could then be made to apply this model in the most efficient and effective manner, and potentially increase participation. In other settings, other types of care models have been combined with VA-ECHO including asynchronous component and a patient participation component (22).

This program is designed to establish a mentor-mentee relationship between hub and spoke clinicians. Learning and relationship building are expected to be promoted when the specialty care team members possess good interpersonal skills and conduct sessions with professionalism. These qualifications are especially important to promote dialogue and open discussion using the video teleconferencing system and should be considered when building new programs.

This study has some limitations including the sole use of email contact for recruitment. Providers and staff receive a large volume of email making overlooking an email relatively prevalent. It may not have always been clear to all recipients why they were being contacted. Respondents agreeing to be interviewed had participated in established VA-ECHO programs and primarily reported on positive attributes of the program. Participants from other programs may have not responded to emailed interview requests. Due to these limitations and the fact that participation was restricted to VHA staff, findings may not be generalizable to non-VHA settings as the VHA is unique within the United States being the largest health care delivery system.

### Recommendations and Future Studies

Future studies could examine retention rates for VA-ECHO participants and explore this program from the perspective of the patient. Consistent with prior studies (6, 7, 18) respondent participants in this study reported positive experiences with the program but how that may or may not influence retention has not been explored. Respondents shared that attendance was feasible when leadership was assured that their participation would not interfere with seeing patients, yet no participants reported monitoring clinic utilization for any potential effects. One spoke site discontinued participation in the program upon realizing that the program focus was not applicable to their specialized clinic. This site, as most, learned about the program by word of mouth. A better fit of participants could be obtained with a more formalized outreach. Finally, patients receiving pain care from providers participating in VA-ECHO were interviewed as part of the overall quality improvement evaluation and these findings will be reported separately.

## Conclusion

This evaluation provides indications that the pain management VA-ECHO program is successful in meeting the needs of the primary care staff to improve pain care for Veterans. Tele-mentoring-based programs are growing in use to educate primary care staff. Based on respondents' comments, the program format fostered improvements in confidence, knowledge and skills as well as learning and implementation of critical soft skills specific to providing pain care to Veterans. The format, whether video-based or audio only, filled a critical gap in participants' education: Learning how to talk to patients about their pain care. More in-depth analysis of how providers learn to have those difficult conversations will require further investigation.

## Data Availability Statement

The datasets generated for this study are available on request to the corresponding author.

## Author Contributions

SB, KS, LS, and RH contributed to the study design, data collection, data analysis, and writing the manuscript. DAu, PH, and DAr were primarily responsible for the design of the larger evaluation of the VA-ECHO program and contributed to the writing of this manuscript.

## Conflict of Interest

DAu receives remuneration from Novarits Inc. for participation on a data safety monitoring board. He serves as a Deputy Editor for the Annals of the American Thoracic Society and is a member of the Exam Committee for the American Board of Internal Medicine Pulmonary Board, for which he receives remuneration. The remaining authors declare that the research was conducted in the absence of any commercial or financial relationships that could be construed as a potential conflict of interest.

## References

[1] Kerns RD Otis J Rosenberg R Reid MC. Veterans' reports of pain and associations with ratings of health, health-risk behaviors, affective distress, and use of the healthcare system. J Rehabil Res Dev. (2003) 40:371–79. 10.1682/JRRD.2003.09.0371 15080222

[2] Mezei L Murinson BB. Pain education in North American medical schools. J Pain. (2011) 12:1199–208. 10.1016/j.jpain.2011.06.006 21945594 PMC13235945

[3] Arora S Thornton K Murata G Deming P Kalishman S Dion D. Outcomes of treatment for hepatitis C virus infection by primary care providers. N Engl J Med. (2011) 364:2199–207. 10.1056/NEJMoa1009370 21631316 PMC3820419

[4] Palaganas EC Sanchez MC Molintas MP Caricativo RD Reflexivity in qualitative research: a journey of learning. Q Rep. (2017) 22:426–38. Retrieved from https://nsuworks.nova.edu/tqr/vol22

[5] Holtrop JS Rabin BA Glasgow RE. Qualitative approaches to use of the RE-AIM framework: rationale and methods. BMC Health Serv Res. (2018) 18:177. 10.1186/s12913-018-2938-8 29534729 PMC5851243

[6] Stevenson L Ball S Haverhals LM Aron DC Lowery J. Evaluation of a national telemedicine initiative in the veterans health administration: factors associated with successful implementation. J Telemed Telecare. (2018) 24:168–78. 10.1177/1357633X16677676 27909208

[7] SayreGGHaverhalsLMBallSStevensonLBattagliaCAronDC. Adopting SCAN-ECHO: the providers' experiences. Healthcare. (2017) 5:29–33. 10.1016/j.hjdsi.2016.04.00628668200

[8] Hill CE Knox S Thompson BJ Williams EN Hess SA Ladany N Consensual qualitative research: an update. J Couns Psychol. (2005) 52:196–205. 10.1037/0022-0167.52.2.196

[9] Hill CE Thompson BJ Williams EN A guide to conducting consensual qualitative research. Couns Psychol. (1997) 25:517–72. 10.1177/0011000097254001

[10] Jansen BDW Brazil K Passmore P Buchanan H Maxwell D McIlfatrick SJ Evaluation of the impact of telementoring using ECHO© technology on healthcare professionals' knowledge and self-efficacy in assessing and managing pain for people with advanced dementia nearing the end of life. BMC Health Ser Res. (2018) 18:228 10.1186/s12913-018-3032-y PMC5879835 29606132

[11] Furlan AD Zhao J Voth J Hassan S Dubin R Stinson JN. Evaluation of an innovative tele-education intervention in chronic pain management for primary care clinicians practicing in underserved areas. J Telemed Telecare. (2018) 25:484–92. 10.1177/1357633X18782090 29991316

[12] Ball S Wilson B Ober S Mchaourab S. SCAN-ECHO for pain management: implementing a regional telementoring training for primary care providers. Pain Med. (2018) 19:262–68. 10.1093/pm/pnx122 28525633

[13] Eaton LH Godfrey DS Langford DJ Rue T Tauben DJ Doorenbos AZ Telementoring for improving primary care provider knowledge and competence in managing chronic pain: a randomised controlled trial. J Telemed Telecare. (2018) 27:1–8. 10.1177/1357633X18802978 PMC10802791 30261805

[14] Katzman JG Comerci G Jr Boyle JF Duhigg D Shelley B Olivas C. Innovative telementoring for pain management: project ECHO pain. J Contin Educ Health Prof . (2014) 34:68–75. 10.1002/chp.21210 24648365

[15] Frank JW Carey EP Fagan KM Aron DC Todd-Stenberg J Moore BA. Evaluation of a telementoring intervention for pain management in the veterans health administration. Pain Med. (2015) 16:1090–100. 10.1111/pme.12715 25716075

[16] Zhou C Crawford A Serhal E Kurdyak P Sockalingam S. The impact of project ECHO on participant and patient outcomes: a systematic review. Acad Med. (2016) 91:1439–61. 10.1097/ACM.0000000000001328 27489018

[17] Katzman JG Qualls CR Satterfield WA Kistin M Hofmann K Greenberg N. Army and navy ECHO pain telementoring improves clinician opioid prescribing for military patients: an observational cohort study. J Gen Intern Med. (2018) 34:387–95. 10.1007/s11606-018-4710-5 30382471 PMC6420488

[18] Ní Cheallaigh C O'Leary A Keating S Singleton A Heffernan S Keenan E Hepatitis C outcomes research network. Telementoring with project ECHO: a pilot study in Europe. BMJ Innov. (2017) 3:144–51. 10.1136/bmjinnov-2016-000141 PMC5754872 29445515

[19] Carlin L Zhao J Dubin R Taenzer P Sidrak H Furlan A. Project ECHO telementoring intervention for managing chronic pain in primary care: insights from a qualitative study. Pain Med. (2018) 19:1140–46. 10.1093/pm/pnx233 29036381 PMC5998945

[20] Katzman JG Galloway K Olivas C McCoy-Stafford K Duhigg D Comerci G. Expanding health care access through education: dissemination and implementation of the ECHO model. Mil Med. (2016) 181:227–35. 10.7205/MILMED-D-15-00044 26926747

[21] Fischer SH Rose AJ McBain RK Faherty LJ Sousa J Martineau M Evaluation of Technology-Enabled Collaborative Learning and Capacity Building Models: Materials for a Report to Congress. Santa Monica, CA: RAND Corporation (2019). Available online at: https://www.rand.org/pubs/research_reports/RR2934.html.

[22] Wood BR Mann MS Martinez-Paz N Unruh KT Annese M Spach DH. Project ECHO: telementoring to educate and support prescribing of HIV pre-exposure prophylaxis by community medical providers. Sex Health. (2018) 15:601–5. 10.1071/SH18062 30318034

